# Spontaneous Brainstem Hemorrhagic Stroke in the Setting of Novel Coronavirus Disease 2019 – A Case Report

**DOI:** 10.7759/cureus.10809

**Published:** 2020-10-05

**Authors:** Gabriel Flores, Jay I Kumar, Elliot Pressman, Jayson Sack, Puya Alikhani

**Affiliations:** 1 Neurosurgery & Brain Repair, University of South Florida, Tampa, USA

**Keywords:** 2019 novel coronavirus disease, case report, covid-19, intracerebral hemorrhage, stroke

## Abstract

Coronavirus disease 2019 (COVID-19) is caused by severe acute respiratory syndrome coronavirus 2 (SARS-CoV-2) and has become a global pandemic. This disease has been shown to affect various organ systems, including the cerebrovascular system with sequelae still not completely uncovered. We present an unusual case of extensive brainstem intraparenchymal hemorrhage in a patient with COVID-19 to caution readers of this possible complication in patients positive for COVID-19. In this report, we outline the clinical presentation of a 40-year-old male who developed severe coughing and sneezing before presenting to the emergency department with confusion, somnolence, and respiratory distress. CT head without contrast revealed extensive pontine and midbrain hemorrhage with intraventricular extension and early hydrocephalus. Neurological examination revealed pinpoint, minimally reactive pupils, withdrawal to painful stimuli in the right hemibody, left hemibody paresis, and intact left corneal, cough, and gag reflexes. MRI and MRA brain revealed no evidence of an underlying vascular lesion. Over the next two days, the patient had worsening multiorgan failure and hypoxemia without intracranial hypertension. He remained too unstable to undergo cerebral angiogram. On hospital day four, his neurological examination deteriorated to quadriparesis and only cough and gag reflexes remaining intact after which his family opted for comfort measures only. In summary, a potential increased risk of intracerebral hemorrhage adds to the complexity of management of patients with COVID-19. This is especially true in those who have violent sneezing or coughing, or those who are on anticoagulation or antiplatelet therapy.

## Introduction

Since initial reports in December 2019 from Wuhan, China, the coronavirus disease 2019 (COVID-19) caused by severe acute respiratory syndrome coronavirus two (SARS-CoV-2) has become a global pandemic. While primarily a respiratory disease, SARS-CoV-2 is also known to affect multiple disparate organ systems, including the cerebrovascular system, however, this is typically in the setting of an ischemic event [1]. Some case reports have been published so far about hemorrhages in the cerebrovascular system in the setting of other risk factors, however to our knowledge, there are no reports on patients with hemorrhage in the absence of risk factors [1-7]. We present a unique case of extensive brainstem intraparenchymal hemorrhage in a patient with COVID-19 to caution readers of this possible complication during this pandemic, even in patients without prior risk factors for hemorrhage.

## Case presentation

**Figure 1:**
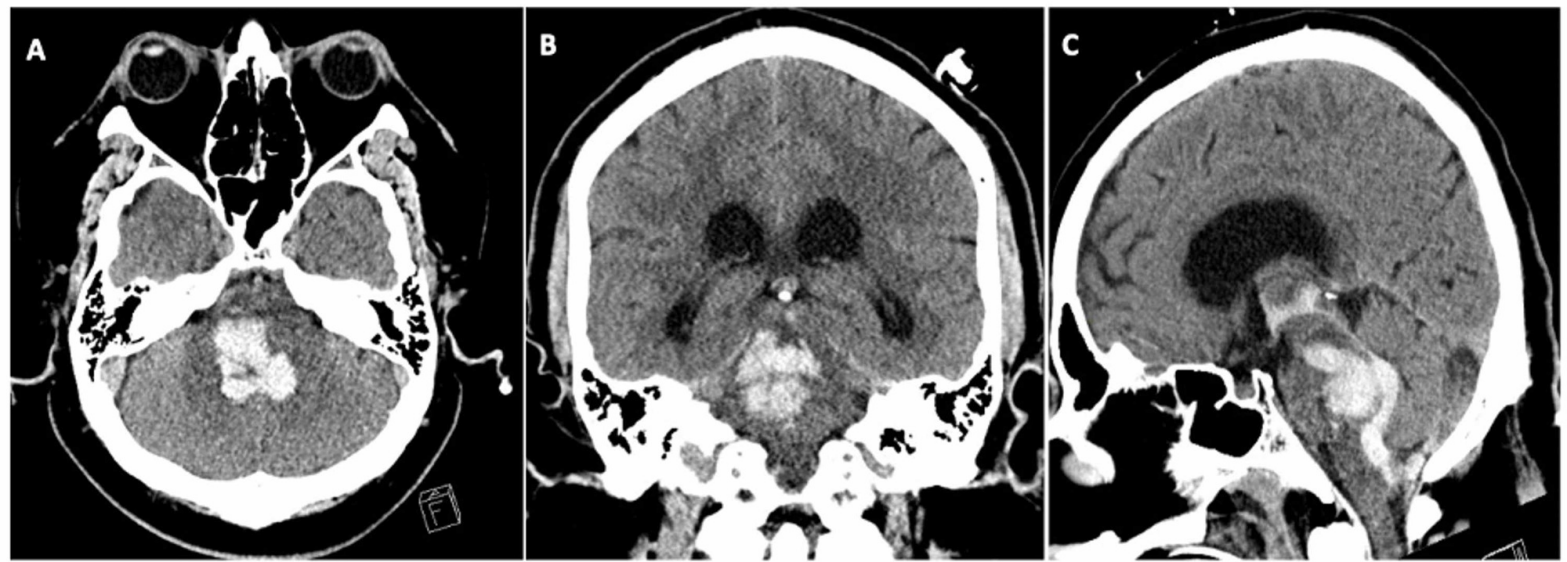
CT head without contrast revealed extensive pontine and midbrain hemorrhage with intraventricular extension involving the third and fourth ventricles and early hydrocephalus (A) Axial image at the level of the pons (B) Coronal image centered at largest diameter of intraparenchymal hematoma (C) Sagittal image showing hemorrhage extending to 4th and 3rd ventricles

Our patient is a 40-year-old male with past medical history of obesity, hypertension, and type two diabetes mellitus who developed severe coughing and sneezing before presenting to the emergency department with confusion, somnolence, and respiratory distress. He was hypertensive (167/87 mmHg) with tachycardia and oxygen saturation (SaO2) of 88%. He was intubated for respiratory protection. A CT head without contrast revealed extensive pontine and midbrain hemorrhage with intraventricular extension into the third and fourth ventricles with early hydrocephalus (Figure 1). He underwent emergent transfer to our tertiary center for neurosurgical evaluation. Neurological examination revealed pinpoint, minimally reactive pupils, withdrawal to painful stimuli in the right hemibody, left hemibody paresis, and intact left corneal, cough, and gag reflexes (Glasgow Coma Scale Eyes one, Verbal zero, Motor four). An external ventricular drain was placed with an opening intracranial pressure (ICP) of 10 mmHg. A chest radiograph revealed bilateral pulmonary congestion, infiltrates, and cardiomegaly. SaO2 remained at 88% on 80% O2. Chest radiograph revealed bilateral pulmonary congestion, infiltrates, and cardiomegaly.

**Figure 2:**
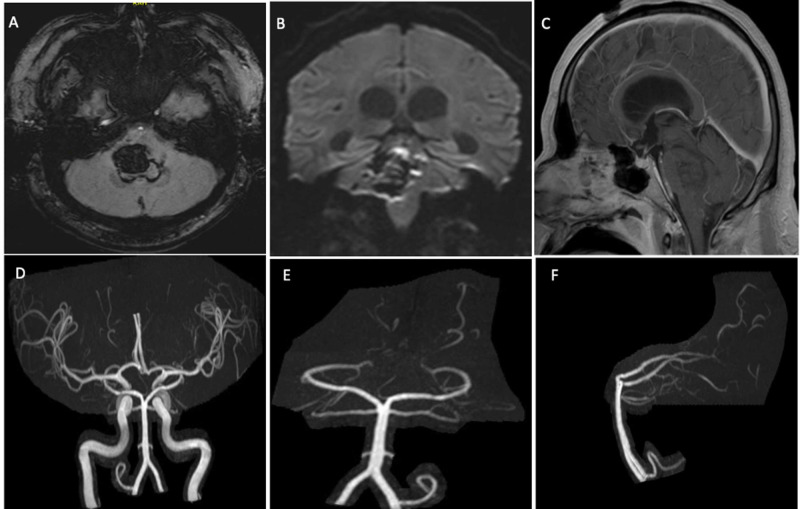
Brain MRI with and without contrast showing brainstem hematoma centered in the pons, just to the right of midline with extension into the right middle cerebral peduncle associated with intraventricular extension (A) Axial susceptibility-weighted image at the level of the pons showing the brainstem hematoma (B) Coronal diffusion-weighted image at the level of the brainstem showing the largest diameter of intraparenchymal hematoma (C) Sagittal post-gadolinium T1 image at the midline demonstrating the lack of contrast enhancement at the hematoma (D) Anteroposterior MRA reconstruction showing both the anterior and posterior circulations demonstrating a normal circle of Willis (E) Anteroposterior MRA reconstruction showing the posterior circulation with no evidence of arteriovenous malformation (F) Lateral MRA reconstruction showing the posterior circulation with no evidence of arteriovenous malformation

Initial labs were notable for elevated inflammatory markers and D-dimer. Per our hospital’s COVID-19 protocol, the patient was placed in a negative pressure room, and a SAR-CoV-2 rapid polymerase chain reaction (PCR) test returned positive. Exposed personnel followed the hospital’s exposure protocol for testing and self-isolation in accordance with Centers for Disease Control recommendations. MRI brain with and without contrast and MRA brain without contrast revealed no evidence of an underlying vascular lesion (Figure 2). On hospital day two, the patient had worsening respiratory distress with multi-organ failure. The patient did not qualify for remdesivir or convalescent plasma treatment by the hospital’s protocol. Over the next two days, the patient had worsening multiorgan failure and hypoxemia without intracranial hypertension. He remained too unstable to undergo cerebral angiogram for further evaluation. On hospital day four, his neurological examination deteriorated to a Glasgow Coma Scale of three with only cough and gag reflexes remaining intact. The family was counseled and opted for comfort measures only.

## Discussion

This report demonstrates the potential for intracranial hemorrhage in patients with COVID-19 despite no apparent bleeding diathesis. The strength of this report lies in its novelty and in our patient’s lack of previously associated risk factors or provocative factors for intracranial hemorrhage such as hypertensive emergency, anti-coagulation medication use, or anti-platelet medication use, though our patient was obese and had diagnosed diabetes mellitus. The main limitation in this report was our inability to obtain a diagnostic cerebral angiogram to further evaluate the patient’s vascular pathology.

Neurological complications are not uncommon in patients with COVID-19 [1]. Due to concern for systemic small vessel thrombosis, many COVID-19 patients have been anti-coagulated, increasing risk of cerebral hemorrhage [2]. Another study reported intracerebral hemorrhage in 33 patients (22 were on anticoagulation, three on antiplatelets, two with thrombocytopenia and one with idiopathic systemic hemorrhage) [3].

Intracerebral hemorrhage without a bleeding diathesis has been reported only rarely in COVID-19 patients [4]. One was a 79-year-old man who did not receive anticoagulants and had normal platelet levels [5]. Another patient was a 66-year-old woman who had been revived from a cardiac arrest prior to hospitalization [6]. A third patient was reported in a series of 214 COVID-19 patients from Wuhan [1], and five more were reported in a series of 26 COVID-19 patients in Italy [7].

The pathogenesis of intracerebral hemorrhage in our patient is unclear. Severe cough was reported to cause cerebral hemorrhage in patients with whooping cough as early as 1885 [8]. The SARS-CoV-2 virus has been demonstrated to bind angiotensin converting enzyme two (ACE2) on endothelial surfaces [9]. This binding inhibits the local protective function of ACE2 and likely compromises endothelial integrity [10]. It is possible that a sudden rise in ICP caused by violent coughing and sneezing could have disrupted the already-compromised vascular endothelium, causing cerebral hemorrhage.

## Conclusions

Increased risk of intracerebral hemorrhage adds to the complexity of management of patients with COVID-19. Clinicians should be aware that in patients with COVID-19, especially in those who have additional violent sneezing or coughing, that they be additionally predisposed to intracerebral hemorrhage. Our report details such a case in a patient not on any anticoagulation or antiplatelet therapy, however, the risk is likely even higher in patients also on anticoagulation or antiplatelet therapy. We believe this additional risk is due to the virus’s ability to bind to angiotensin converting enzyme two on endothelial surfaces.
